# Validation of the Vitiligo noticeability scale: a patient-reported outcome measure of Vitiligo treatment success

**DOI:** 10.1186/1745-6215-16-S2-P68

**Published:** 2015-11-16

**Authors:** Jonathan Batchelor, Wei Tan, Selina Tour, Andrian Yong, Alan Montgomery, Kim Thomas

**Affiliations:** 1 Centre of Evidence Based Dermatology, University of Nottingham, Nottingham, England; 2 Nottingham Clinical Trials Unit, University of Nottingham, UK; 3 Norfolk and Norwich University Hospitals NHS foundation Trust, Norwich, England, UK

## Introduction

Outcome assessment in vitiligo studies is inconsistent and development of a core outcome set is underway. Percentage repigmentation is commonly measured, but patient-reported outcomes measures are rare. The Vitiligo Noticeability Scale (VNS) is a new patient-reported measure of treatment success. The aim of this study was to assess the construct validity, acceptability and interpretability of the VNS and identifies possible predictors of treatment success when using the scale.

## Methods

Using an online questionnaire comprising 39 image pairs of vitiligo pre- and post-treatment, 101 participants with vitiligo assessed treatment success using a binary global scale and the VNS scale. 33 clinicians performed the same two assessments, together with percentage repigmentation (> 75% defined as success). Agreement between respondents within the same scale and between different scales were assessed using kappa statistics with bootstrapping. Association between VNS score and image/respondent characteristics was estimated using logistic regression.

## Results

VNS scores were associated with both patient- and clinician-reported global treatment success (k = 0.54 and k = 0.47 respectively). Percentage repigmentation showed a weaker association with treatment success (k=0.39 for patient and 0.29 for clinician). VNS scores of 4 or 5 can be interpreted as representing treatment success and 3 as partial success. Images showing post-treatment hyperpigmentation were less likely to be rated as successful.

## Conclusions

The VNS is a valid patient-reported measure of vitiligo treatment success, which is suitable for use in clinical trials and clinical record keeping. Allowing time for resolution of residual hyperpigmentation post-treatment, before assessing VNS score, may be advisable.

